# Recent advances in the *Zymoseptoria tritici*–wheat interaction: insights from pathogenomics

**DOI:** 10.3389/fpls.2015.00102

**Published:** 2015-02-24

**Authors:** Megan C. McDonald, Bruce A. McDonald, Peter S. Solomon

**Affiliations:** ^1^Plant Sciences Division, Research School of Biology, The Australian National University, Canberra, ACT, Australia; ^2^Plant Pathology Group, Institute of Integrative Biology, ETH Zurich, Zurich, Switzerland

**Keywords:** wheat pathogen, *Mycosphaerella graminicola*, necrotroph, effector, RNA-seq, comparative genomics

## Abstract

We examine the contribution of next generation sequencing (NGS) to our understanding of the interaction between the fungal pathogen *Zymoseptoria tritici* and its wheat host. Recent interspecific whole genome comparisons between *Z. tritici* and its close relatives provide evidence that *Z. tritici* has undergone strong adaptive evolution, which is attributed to specialization by *Z. tritici* on wheat. We also assess the contribution of recent RNA sequencing datasets toward identifying pathogen genes and mechanisms critical for disease. While these studies have yet to report a major effector gene, they illustrate that assembling reads to the reference genome is a robust method to identify fungal transcripts from *in planta* infections. They also highlight the strong influence that the wheat cultivar has on effector gene expression. Lastly, we suggest future directions for NGS-guided approaches to address largely unanswered questions related to cultivar and lifecycle dependent gene expression and propose that future experiments with *Z. tritici* be conducted on a single wheat cultivar to enable comparisons across experiments.

## INTRODUCTION

*Zymoseptoria tritici* (P. Crous; syn. *Mycosphaerella graminicola, Septoria tritici*) causes the wheat disease *Septoria tritici* blotch (STB). STB is globally distributed and was recently identified as the most yield-reducing disease in European countries with intensive wheat production (Jørgensen et al., 2014). STB is managed mainly through fungicide applications and to a lesser extent by resistance breeding (Goudemand et al., 2013). Up to 18 loci associated with plant resistance have been identified and incorporated into wheat breeding programs, providing some quantitative resistance to the disease (Orton et al., 2011; Tabib Ghaffary et al., 2012). But the pathogen has proven adept at overcoming plant resistance (Cowger et al., 2000) and evolving fungicide resistance (Cools and Fraaije, 2008; Torriani et al., 2009), therefore new tools and approaches are needed to develop an integrated disease management strategy.

*Zymoseptoria tritici* exhibits a long symptomless phase, typically lasting 8–11 days post infection (dpi), during which the fungus is hypothesized to derive nutrition from the plant without eliciting defense responses. At 8–11 dpi, the host mesophyll cells suddenly collapse, releasing their contents and allowing *Z. tritici* to proliferate rapidly throughout the necrotic tissue (Kema et al., 1996; Duncan and Howard, 2000). Several gene-for-gene interactions between *Z. tritici* and wheat (*Triticum aestivum*) have been hypothesized (Kema et al., 2000; Brading et al., 2002; Goodwin, 2007; Jørgensen et al., 2014), but none of the genes conferring virulence in the fungus or resistance in wheat have been cloned. The detection of gene-for-gene interactions implies that *Z. tritici* should encode proteins or metabolites that mediate genotype-specific interactions (Brading et al., 2002).

The reference genome for *Z. tritici*, based on the Dutch isolate IPO323, is among the most complete fungal genome sequences available (Goodwin et al., 2011). The genome contains 13 core chromosomes and eight accessory chromosomes (ACs), the highest number of ACs reported in filamentous fungi (Mehrabi et al., 2007; Goodwin et al., 2011). This genome has now been incorporated into the online database EnsemblFungi, which includes standardized gene file formats and other data useful for any next generation sequencing (NGS) project (Flicek et al., 2014). The completeness of the IPO323 assembly makes *Z. trtici* one of the most approachable fungal pathogens for conducting NGS experiments. In this review, we highlight several of the most recent comparative genomic and RNA-sequencing (RNA-seq) experiments and examine their contribution toward understanding the *Z. tritici*–wheat interaction. We seek to identify both the strengths and weaknesses of each approach in order to guide future NGS experiments toward more multifaceted and cross-disciplinary designs.

## INTERSPECIFIC COMPARATIVE GENOMICS AND THE ROLE OF ACCESSORY CHROMOSOMES

The evolutionary history of *Z. tritici* is arguably the most comprehensively understood for any fungal pathogen. Earlier studies using coding and microsatellite loci indicated that the center of diversity for the pathogen coincides with its host *Triticum aestivum* (bread wheat) in the ancient Fertile Crescent (Banke et al., 2004; Stukenbrock et al., 2007). Population genetic analyses of fungi collected from wild grasses growing in close proximity to cultivated wheat in Iran revealed two sister species of *Z. tritici*. These close relatives, now named *Zymoseptoria pseudotritici* and *Zymoseptoria ardabiliae* (syn. S1 and S2 in earlier publications, respectively), were used for extensive genomic comparisons with *Z. tritici* (Stukenbrock et al., 2010, 2011). Importantly, *Z. pseudotritici* and *Z. ardabiliae* produce much lower levels of infection on domesticated wheat compared to *Z. tritici*. Genome comparisons of several strains of all three species sampled from the same location revealed a large set of shared genes (Stukenbrock et al., 2011). These genes provided strong evidence of adaptive evolution, measured through ratios of non-synonymous/synonymous mutations, occurring in *Z. tritici* when compared to the other species (Stukenbrock et al., 2011). Another important observation was elevated rates of non-synonymous substitutions in genes containing signal peptides in *Z. tritici* (Stukenbrock et al., 2011). This indicated that the genes most likely to encode secreted proteins that interact with the host or environment have undergone adaptive evolution, providing important support for the hypothesis that the effector proteins in *Z. tritici* are likely to be secreted.

Much speculation has been directed toward the role of *Z. tritici’s* ACs (Croll and McDonald, 2011; Croll et al., 2013). Unlike those of *Fusarium oxysporum* or *Nectria haematococca*, *Z. tritici* ACs have not been shown to confer host-specific virulence (Miao et al., 1991; Ma et al., 2010). *Z. tritici’s* ACs also appear to be inherited from a common ancestor as both *Z. pseudotritici* and *Z. ardabiliae* were found to carry ACs syntenic with *Z. tritici* (Stukenbrock et al., 2011). While there are clear paralogs between the core and accessory genomes, transfer of syntenic chromosomal regions from the core to the accessory genome has not been detected (Croll and McDonald, 2011; Goodwin et al., 2011). In a recent RNA-seq study, Kellner et al. (2014) investigated the origin of ACs genes after observing that many were differentially expressed between *in planta* and axenic culture conditions. After grouping all genes in the genome into families they found a significantly higher proportion of unique genes on ACs. For the small proportion (∼4–6%) of AC genes that did belong to gene families, phylogenetic analysis revealed that they form their own monophyletic group with orthologous dothidiomycete genes, suggesting ancient duplication events. Comparisons of core and ACs uncovered an enrichment of predicted miRNA loci on the ACs that are presumed to regulate expression of genes on ACs (Goodwin et al., 2011; Kellner et al., 2014). Whilst preliminary, these data are consistent with sophisticated regulation of genes located on ACs. This hypothesis was supported by RNA-seq data from Kellner et al. (2014) and Rudd et al. (2015) who showed that genes on ACs are expressed on average 13-fold and 4- to 5-fold less, respectively, than genes on core chromosomes.

Taken together, these comparative genomic datasets indicate that *Z. tritici* is a recently emerged and rapidly evolving pathogen that has become highly specialized to infect wheat compared to its close relatives. But despite access to genome sequences of these relatives, comparative genomics has yet to reveal any proven effector genes even though several hundred genes are unique to each species. A major remaining gap in *Z. tritici* genomics is extensive intra-specific genome comparisons. To date, re-sequencing of *Z. tritici* was limited to two Iranian isolates used for interspecific comparative genomics (Stukenbrock et al., 2011) and nine Swiss isolates used to characterize ACs, intra-specific intron polymorphisms, and selection operating on plant cell wall degrading enzymes (CWDEs; Torriani et al., 2011; Brunner et al., 2013; Croll et al., 2013). While Stukenbrock et al. (2010) investigated genes shared between *Z. tritici* and *Z. pseudotritici*, genes showing presence/absence polymorphisms among re-sequenced *Z. tritici* genomes have not been reported. Similarly, there has not been a coordinated re-sequencing effort using isolates that show differential phenotypes on wheat cultivars with known resistance alleles, despite the availability of appropriate isolates known to exhibit extensive phenotypic and chromosomal differences (IPO94269, IPO04241, IPO95952; Kema et al., 2000; Brading et al., 2002; Mehrabi et al., 2007; Wittenberg et al., 2009). We propose that more extensive intraspecific re-sequencing is needed to assess whether there is evidence of pathotype-specific alleles (SNPs), genes or larger genomic regions linked with virulence.

## IDENTIFYING VIRULENCE GENES

Before the release of the reference genome, several genes orthologous to verified pathogenicity genes in other fungi were functionally investigated through genetic knockouts. Many of these targeted genes were conserved signaling proteins and their functions in *Z. tritici* were recently reviewed (see Orton et al., 2011). An important advance since the publication of this review shows Mg3LysM (the only effector identified in *Z. tritici* that is required for virulence and when functionally inactivated is indistinguishable from *in vitro* wild type growth; Marshall et al., 2011), suppresses PAMP-triggered immunity (PTI) mediated by the chitin recognizing CERK1 receptor kinase and CEBiP receptor-like protein (Lee et al., 2014). Virus-induced gene silencing of CERK1 and CEBiP partially restored the pathogenicity of the avirulent Δ*Mg3LysM* isolate (Lee et al., 2014). This is the first evidence that *Z. tritici* secretes a protein that actively suppresses plant defenses during infection.

The important role of *Mg3LysM* in pathogenicity was discovered through homology to known virulence genes in other plant pathogens. More recently, the discovery of novel effectors in *Z. tritici* has been attempted using RNA-seq. Yang et al. (2013) analyzed gene expression on a susceptible wheat cultivar at 4, 10, and 13 dpi. The authors first assembled the transcriptome *de novo* including both fungal and plant reads and subsequently separated the fungal and plant reads by BLAST analysis, resulting in the identification of 1829 fungal transcripts (Yang et al., 2013). The number of identified transcripts in this study is extremely low compared to two other *in planta* RNA-seq experiments where fungal gene expression was compared in infected tissue and axenic culture (Kellner et al., 2014; Rudd et al., 2015). Kellner et al. (2014) identified over 7000 fungal transcripts at 6 dpi in both infected wheat and *B. distachyon* plants, while Rudd et al. (2015) identified between 9,236 and 10,485 transcripts with sampling times at 1, 4, 9, 14, and 21 dpi. Kellner et al. (2014) identified 156 genes that were significantly more highly expressed *in planta* when compared with axenic culture. Unfortunately, they do not present any functional data that show these genes play a role in virulence. Rudd et al. (2015) identified 115 putative secreted proteins with peak expression at 9 dpi, coincident with the appearance of necrotic lesions. Of these, five were chosen for functional analysis though gene deletion. None of the mutant strains showed reduced pathogenicity on wheat. While both studies identified several hundred potential effector genes, the negative functional results in Rudd et al. (2015) illustrate the great challenge that redundancy brings to validating hypothesized effector genes in this pathogen. Coupled with the time-consuming and low transformation efficiency of *Z. tritici* with *Agrobacterium tumefaciens*, this apparent redundancy poses the biggest challenge to the ability of RNA-seq to identify effector candidates.

## PROFILING EXPRESSION OF GENE FAMILIES AND METABOLIC PATHWAYS

Important insights from RNA-seq are not limited to the discovery of virulence genes. Brunner et al. (2013) investigated the expression of plant CWDEs throughout infection. Degraded plant oligosaccharides are known to induce innate plant immunity (Esquerré-Tugayé et al., 2000; Vorwerk et al., 2004). The *Z. tritici* reference genome isolate IPO323 contained significantly fewer CWDEs compared to other plant pathogenic fungi, which led to the hypothesis that the scarcity of these enzymes may be linked to its prolonged latent period (Goodwin et al., 2011). Using RNA-seq, Brunner et al. (2013) quantified the *in planta* expression of all CWDEs at 7, 13, and 56 dpi. They reported that 28 of the 48 CWDE genes showed life cycle stage specific expression (Brunner et al., 2013). Among these 28 genes, only three (protein IDs: 18212, 106779, 94846) had their highest expression at 7 dpi, prior to symptom development. These data show that plant CWDE expression is tightly regulated by *Z. tritici*, particularly during the asymptomatic phase of infection and was largely confirmed in an independent RNA-seq experiment by Rudd et al. (2015). This result lends support to the earlier hypothesis by Goodwin et al. (2011) that tight regulation or scarcity of these genes or their products during infection is critical to avoiding plant recognition.

RNA-seq also sheds light on *Z. tritici’s* early source of nutrients during the prolonged latent period. Rudd et al.’s (2015) extensive deep RNA-seq experiment provides strong evidence that *Z. tritici’s* main energy source during the latent period (1–4 dpi) comes from stored lipids. While lipid metabolism is dominant during early plant colonization, the fungus transitions at 9 dpi to express large numbers of secreted proteases that increase in expression from 14 to 21 dpi along with CWDE expression, indicating a shift to more complex carbohydrate metabolism at the late stages of infection. Previously, Keon et al. (2005, 2007) used a micro-array to characterize nutrition-dependent gene expression in both nutrient rich and nutrient poor conditions *in vitro* and *in planta*. They note, however, that they were unable to estimate fungal gene expression at 6 dpi due to very low biomass and poor hybridization. RNA-seq has eliminated the biomass constraint from early *in planta* time-points.

Below we discuss potential design improvements for future NGS experiments to address unexplored facets of the *Z. tritici*–wheat interaction.

## FUTURE NGS EXPERIMENTS

### CULTIVAR DEPENDENT GENE EXPRESSION

When comparing gene expression between the published NGS and quantitative PCR (qPCR) datasets, some inconsistencies become immediately apparent. For example, Marshall et al. (2011) found via qPCR that *Mg3LysM* was up-regulated during early infection *in planta* but not *in vitro*, while Kellner et al. (2014) did not find differential expression *in planta* compared to axenic culture using RNA-seq. Similarly Yang et al. (2013) found that *Mg3LysM* was most highly expressed at 13 dpi. This contradicts Marshall et al. (2011) who found high expression at days four and nine but little expression at day 14. Though each of these studies used the IPO323 isolate, they each used different susceptible wheat lines, and whilst disease developed normally on each host, it is likely that different host genotypes will lead to differential pathogen gene expression. This illustrates the need to choose a uniform host genotype in the *Z. tritici* community to enable comparisons of effector searches among labs.

The apparent redundancy in effector gene function (i.e., the suppression of effector triggered immunity) makes it difficult to functionally validate candidate effectors through targeted knockouts (Rudd et al., 2015). An important RNA-seq experiment will be to measure gene expression for the same isolate on both susceptible and resistant cultivars across several time points, preferably both before and after the onset of necrosis. It will also be essential to compare the transcriptomes of isolates that differ in virulence on the same host over time. These experiments would facilitate the identification of highly expressed transcripts during infection and also differences in fungal gene expression during a compatible vs. incompatible interaction. A comprehensive RNA-seq experiment that incorporates all of the afore mentioned variables (several isolates, two cultivars and at least three time points) would enable researchers to begin to tease apart genes whose expression is more strongly influenced by host or isolate genotype or alternatively by life cycle stage.

### FINDING MAJOR VIRULENCE GENES

Isolate specific interactions between *Z. tritici* and wheat are commonly reported in both field and genetic mapping studies (Cowger et al., 2000; Brading et al., 2002; Goodwin, 2007; Chartrain et al., 2009; Mehrabi et al., 2014). Yet, the corresponding virulence genes in *Z. tritici* have not been identified. The steadily declining costs of NGS provide the opportunity to conduct large scale genome wide association studies (GWAS) or use quantitative trait locus (QTL) mapping approaches specifically aimed at identifying genetic regions associated with isolate specific virulence. The QTL mapping approach has already been successfully applied in *Z. tritici* to identify 16 novel candidate genes and a quantitative trait nucleotide in a known gene associated with melanization (Lendenmann et al., 2014). Previously, marker development was both time-consuming and expensive and resulted in a very sparse genetic map. But techniques such as reduced representation genome sequencing, RAD-seq and other techniques provide a dense genetic map across the entire genome at minimal cost per individual (Davey et al., 2011; Lendenmann et al., 2014). These techniques should be extremely powerful in identifying loci significantly associated with virulence that could in turn provide molecular biologists with candidate genes for functional validation. An opportunity also lies in comparing the candidate virulence loci identified through QTL mapping and GWAS with candidate virulence genes identified through RNA-sequencing. Significant overlap between these independent approaches could further prioritize genes for functional characterization, either through genetic knockouts or heterologous expression.

## PERSPECTIVES

Despite extensive sequencing efforts, neither comparative genomics nor transcriptomics have yet functionally identified any genes that are required for virulence. Is this because these genes are not true effectors? Or has this pathogen layered its attack mechanisms so effectively that the loss of one gene function is easily compensated by the action of another? The resistance loci mapped in wheat indicate that this should not be the case for all genes. Hammond-Kosack and Rudd (2008) proposed that the mechanisms leading to a compatible interaction between *Z. tritici* and its host are disparate from simplistic models whereby necrotrophs simply secrete large quantities of CWDEs and other lytic enzymes to mechanically break open plant cells. Instead they propose a model in which *Z. tritici*, like other necrotrophic pathogens, utilizes the pre-programmed defense pathways in wheat to induce necrosis, essentially tricking the plant into killing itself (Hammond-Kosack and Rudd, 2008; Deller et al., 2011). NGS datasets, particularly RNA-seq, on an expanded set of isolates displaying differential pathogenicity on a single cultivar would be particularly useful to identify the genes necessary for the successful transition from latency to necrosis. We propose that GWAS and QTL mapping could be useful complementary methods to RNA-seq, by identifying quantitative trait loci that co-localize with candidate genes identified in RNA-seq studies. Identification of the genes required for this transition could provide the key to identifying the plant defense pathways targeted and identify new potential control strategies for this important pathogen.

### Conflict of Interest Statement

The authors declare that the research was conducted in the absence of any commercial or financial relationships that could be construed as a potential conflict of interest.
